# Infectious Disease Management in Resource-Limited Settings

**DOI:** 10.1093/ofid/ofag261

**Published:** 2026-06-09

**Authors:** Carlos Seas

**Affiliations:** Instituto de Medicina Tropical Alexander von Humboldt, Universidad Peruana Cayetano Heredia, Lima, Peru

**Keywords:** Low middle income countries; antimicrobial resistance; HIV; tuberculosis; Chagas disease; traveler′s diarrhea; PCR; undifferetiated fever; Clostridioides difficile

Infectious diseases contribute to 28% of global DALYs, predominantly impacting low- and middle-income countries (LMICs). This supplement highlights various strategies for assessing and alleviating this burden. Antimicrobial stewardship is examined through 3 studies: a multidisciplinary effort to optimize antibiotic use in a pediatric ICU in Botswana; process mapping to identify bottlenecks in treating febrile neutropenia in Uganda; and the use of telehealth to address the shortage of stewardship specialists in rural United States. A study from Peru on a key health determinant, HIV and TB infections, advocates for adopting long-acting HIV PreEP and short rifamycin-based TB treatments. Digital tools, such as AI-powered X-rays for TB detection and mobile clinics providing decentralized mother-to-child HIV prevention in Mozambique, are also explored. Screening and surveillance models for evaluating Chagas disease in rural US and causes of febrile illnesses in a pediatric population in Mozambique highlight the importance of these approaches. Research on traveler's diarrhea identified the strengths and weaknesses of available multiplex diagnostic platforms, and a study in Ethiopia found that fecal microbiota testing is acceptable when paired with education about its rationale. Addressing infectious diseases in LMICs requires integrating simple clinical tools, digital innovations, and active community education to ensure sustainable health improvements.

Infectious diseases pose a significant health challenge in low- and middle-income countries (LMICs). The 2024 burden of disease report indicates that 704 million DALYs worldwide were attributable to 85 pathogens in 2019, accounting for 28% of all DALYs. Notably, the burden of infectious diseases is concentrated primarily in LMICs [1]. This supplement highlights strategies for assessing and alleviating this burden. Topics covered include antimicrobial stewardship, HIV and TB infections, screening and surveillance models for evaluating Chagas disease, causes of febrile illnesses, and research on traveler's diarrhea and fecal microbiota acceptability.

Antimicrobial resistance among common bacterial pathogens in both community and hospital settings is a major global health challenge. Unfortunately, low- and middle-income countries are disproportionately affected by this issue, with the highest levels of resistance observed in Gram-negative and Gram-positive organisms. This supplement features 3 studies that examine several key aspects of the problem.

Bedada and colleagues [2] developed a comprehensive program to improve antimicrobial use in a pediatric surgical ICU in Botswana, where a baseline assessment revealed widespread antimicrobial misuse due to a lack of standardized guidelines, limited knowledge of resistant patterns among bacterial pathogens, and inappropriate antibiotic use. A multidisciplinary team created and implemented evidence-based clinical guidelines and an antimicrobial stewardship program aimed at evaluating antibiotic use following the World Health Organization AwaRe (Access, Watch, Reserve) classification of antibiotics. The results of the intervention were impressive: 92.2% of antibiotic use was appropriate, mainly involving the less extended-spectrum (Access group), and the duration of antibiotic therapy was reduced. This study and others [3] demonstrate that implementing simple measures can significantly influence antimicrobial prescribing practices. The main challenges are the program's sustainability and the integration of modern microbiological methods for detecting prevalent resistance patterns.

In another study, Gulleen and coworkers [4] evaluated the use of process mapping to identify barriers to timely antibiotic administration in LMICs. A 5-step guide, including preparation, data gathering, map generation, analysis, and moving it forward, was developed. Using a case study from the Uganda Cancer Institute on managing febrile neutropenia, the investigators showed how this approach enables visualization of complex care processes and the identification of bottlenecks. Therefore, the investigators demonstrated that process mapping is a valuable tool for clinicians in the developing world and that antimicrobial stewardship programs using it can prioritize local interventions tailored to their specific environments.

The latest study on promoting the rational use of antibiotics comes from the United States. Baus and colleagues [5] explored the potential use of telehealth in rural areas to improve antimicrobial stewardship. These rural areas consume antibiotics at rates similar to those in more urban settings but lack specialized professionals such as infectious disease physicians, pharmacists, nursing staff, and microbiologists. The quick take-home message is that telehealth is a viable and scalable solution for addressing professional shortages in rural settings. Connecting less-served professionals with more advanced professionals to discuss complex cases improves case management. The study provides a roadmap for physicians to advocate for regional networks and serves as a model for LMICs to implement.

Tuberculosis (TBC) and HIV-1 are major health challenges in LMICs. Two studies explore current issues related to these diseases. Quispe et al [6] examined the challenges and opportunities for improving TBC and HIV prevention in South America. The study shows that, despite significant progress in detecting these diseases, both still cause substantial illness and death. Regarding TBC control, none of the countries with the highest incidence in the region are on track to meet the targeted 2030 goals; ongoing epidemics and a lack of sustained political and funding support from local governments partly drive the current crisis. Major obstacles to controlling transmission include fragmented healthcare systems, in which referral systems for better diagnostic and treatment access are nearly impossible to achieve; limited availability of molecular diagnostic methods endorsed by the World Health Organization; and social stigma affecting vulnerable populations. For physicians in LMICs, the paper emphasizes that current efforts are insufficient to reach elimination goals, especially given the volatility of international funding from agencies such as USAID and PEPFAR. The key message for clinicians and policymakers is the urgent need to adopt shorter, rifamycin-based TB preventive therapies and long-acting injectable HIV PrEP to improve adherence. The authors recommend leveraging precision public health and digital tools, such as AI-powered chest X-ray analysis and telemedicine, to close gaps in screening and care. A proposed 10-year roadmap outlines 3 phases: the first 5 years should focus on expanding diagnostic capacity and removing barriers to preventive interventions; the next 5 years should prioritize integrating digital health, artificial intelligence, and preventive units into primary care. Domestic funding after the initial decade should ensure regional autonomy.

Cerini et al present a compelling study [7], ProTeggiMI (an Italian phrase meaning “*protect me*”), which explores a decentralized approach to preventing mother-to-child HIV transmission (PMTCT) in Mozambique's remote regions, where vertical transmission remains very high. Using existing mobile health clinics to implement PMTCT strategies enabled the identification of more women with HIV, helping to integrate them into healthcare and reduce the gestational age at enrollment. The authors highlighted key barriers to care, with adherence being among the most significant. While mobile clinics are an effective tool for reaching underserved and marginalized populations, the sustainability of this intervention depends on consistent services and strong support from patients starting antiretroviral therapy. This study emphasizes the urgent need to bring the clinic directly to patients to overcome access barriers and shows that decentralization of care is not enough unless paired with improved counseling and frequent outreach to reduce the high loss-to-follow-up in less-visited communities. The model evaluated in this study serves as a blueprint for LMICs planning to implement interventions into existing mobile healthcare systems in areas where access to traditional care is limited.

Chagas disease is a chronic illness found exclusively in the Americas. While Central and South American countries have high prevalence rates, migration from these regions to the developed world—driven by ongoing political and economic crises—has expanded its reach into areas where many healthcare providers are unfamiliar with it. The study by Lynn and colleagues [8] evaluated a multi-component approach to detecting Chagas disease in a large US community. The approach included an educational program using grand rounds, a champion initiative involving diverse healthcare staff, consultation with external experts, and a screening program to identify the disease in at-risk individuals. A key message of this study is that educational programs alone are not enough for long-term success. Improving diagnostic rates requires the involvement of the entire healthcare system and its administrative processes to provide access to diagnostic tests for the at-risk population. Active screening of vulnerable populations is an important message to physicians living in LMICs.

The last 3 studies on the supplement address different topics. Toriro and colleagues from the United Kingdom evaluated the diagnostic agreement between 2 multiple PCR platforms (BioFire FilmArray and the Seegene Allplex) among military recruits who contracted traveler's diarrhea in developing countries [9]. The study confirmed the usefulness of these PCRs in quickly detecting enteropathogens, showing agreement in identifying major pathogens such as *Campylobacter* spp., enteroaggregative *Escherichia coli*, and sapovirus, but it demonstrated low performance for 9 other pathogens. FilmArray's sensitivity in detecting certain pathogens needs further improvement.

Infectious causes of febrile illnesses in pediatric populations in LMICs have been the focus of many studies. However, large and long-term cohorts are lacking. Torres-Fernandez and coworkers present data from a 17-year retrospective cohort comprising over 660 000 outpatient encounters and 23 000 hospitalizations among children under 15 years old treated at a district hospital serving several rural clinics in southern Mozambique [10]. As expected, malaria, respiratory, and intestinal infections are the main causes of morbidity and mortality. Interestingly, a steady yearly decrease in the incidence of these infections was observed, which may reflect the implementation of public health interventions. Notable findings also include that neonates and infants with unknown causes had high mortality rates and that malnutrition and HIV significantly contributed to mortality. The study delivers an important message for pediatricians working in resource-limited settings: long-term epidemiological, clinical, and microbiological surveillance programs are essential for identifying causes of febrile illnesses, monitoring changes in incidence over time, and evaluating resistance patterns and modifiable interventions to reduce mortality.

The final study on this supplement is by Shackelford and colleagues [11], who evaluated the knowledge and acceptability of fecal microbiota transplantation among patients, caregivers, and healthcare professionals in Ethiopia. Transplanting fecal microbiota is a standard procedure for treating recurrent *Clostridioides difficile* infection in the developed world. However, its use in less-developed settings has not been thoroughly examined. The study indicates that when patients and healthcare professionals are properly educated about the medical reasons for the procedure, their acceptance increases, but significant safety concerns—such as the risk of acquiring additional infections—remain. A key lesson for clinicians in low- and middle-income countries is that actively educating patients and colleagues about the procedure's potential benefits and risks is crucial for successful implementation.

I hope the reader finds these carefully selected studies on healthcare challenges in LMICs informative and that reading them sparks curiosity and encourages participation in activities that help reduce the burden of the diseases discussed in this special supplement.
